# High-resolution imaging atlas reveals the context-dependent role of pancreatic sympathetic innervation in diabetic mice: Pancreatic sympathetic innervation pattern in diabetic mice

**DOI:** 10.3724/abbs.2024215

**Published:** 2024-12-02

**Authors:** Qingqing Xu, Yuxin Chen, Xinyan Ni, Hanying Zhuang, Shenxi Cao, Liwei Zhao, Leying Wang, Jianhui Chen, Wen Z Yang, Wenwen Zeng, Xi Li, Hongbin Sun, Wei L Shen

**Affiliations:** 1 Biology Science Institutes Chongqing Medical University Chongqing 400016 China; 2 School of Life Science and Technology & Shanghai Clinical Research and Trial Center ShanghaiTech University Shanghai 201210 China; 3 Institute for Immunology and School of Basic Medical Sciences Tsinghua Medicine Tsinghua University Beijing 100084 China

**Keywords:** high-resolution imaging, sympathetic innervation, α/β-cell, chemical sympathetic denervation, diabetes

## Abstract

A better understanding of how sympathetic nerves impact pancreatic function is helpful for understanding diabetes. However, there is still uncertainty and controversy surrounding the roles of sympathetic nerves within the pancreas. To address this, we utilize high-resolution imaging and advanced three-dimensional (3D) reconstruction techniques to study the patterns of sympathetic innervation and morphology in the islets of adult wild-type (WT) and diabetic mice. Our data show that more than ~30% of α/β-cells are innervated by sympathetic nerves in both WT and diabetic mice. Additionally, sympathetic innervated α/β-cells are reduced in diet-induced obese (DIO) mice, whereas sympathetic innervated β-cells are increased in *db*/ *db* mice. In addition, *in situ* chemical pancreatic sympathetic denervation (cPSD) improves glucose tolerance in WT and *db*/ *db* mice but decreases glucose tolerance in DIO mice. *In situ* cPSD also enhances insulin sensitivity in diabetic mice without affecting WT mice. Overall, our findings advance our understanding of diabetes by highlighting the distinctive impact of pancreatic sympathetic innervation on glucose regulation.

## Introduction

Glucose homeostasis is a critical physiological process that maintains the essential energy supply for the human body and prevents the detrimental effects of hyperglycemia or hypoglycemia [1]. This intricate regulation primarily hinges upon the secretion of hormones from the pancreatic islets [2]. Given that islets constitute a small fraction (1%–2%) of the total pancreatic volume [ 3, 4], islets exhibit considerable variations in size [5]. The islets contain α-cells and β-cells that secrete glucagon to increase blood glucose and insulin to lower it. These hormones work together to ensure that glucose levels remain within a narrow, healthy range, supporting the body’s energy needs. Changes in the histomorphology and function of islets correlate with aberrant blood glucose levels [6]. Despite extensive research on the endocrine function of the pancreas, our understanding of the intricate network of nerves that innervate the pancreas and their roles in regulating blood glucose remains limited.

The autonomic nervous system, particularly the sympathetic nervous system, influences the pancreas via visceral nerves originating from the prevertebral abdominal and superior mesenteric ganglia. This neural input is pivotal for the development and maturation of the pancreas [ 7– 9]. Despite the acknowledged importance of sympathetic innervation in pancreatic function, the specific roles of these nerves within the pancreas remain unclear and controversial [10]. Sympathetic innervation currently plays a predominant role in influencing the secretion of islet hormones through the neurotransmitter norepinephrine (NE). NE, released from postganglionic sympathetic fibres or the adrenal medulla, stimulates glucagon secretion by binding to β2-adrenergic receptors on α-cells and inhibits insulin secretion by binding to α2-adrenergic receptors on β-cells [ 11, 12]. The activation of sympathetic nerves through electrical stimulation leads to the release of NE, which mimics the inhibitory effect on glucose-stimulated insulin secretion (GSIS) [13]. Similarly, the administration of exogenous NE or adrenergic receptor agonists replicates the inhibitory effects of sympathetic nerve activation on GSIS [14]. Conversely, adrenergic receptor antagonists counteract the inhibitory effect of sympathetic nerve activation on insulin secretion [15]. In addition, the activation of sympathetic nerves stimulates glucagon secretion and inhibits insulin release, leading to elevated blood glucose levels, which are essential for the fight or flight response [ 16, 17]. However, it has been reported that the developmental loss of sympathetic nerves results in reduced insulin secretion and impaired glucose tolerance in adult mice [7]. To address the existing controversies, we need to manipulate the sympathetic nerves in the pancreas to clarify their roles in glucose metabolism. However, the detailed sympathetic innervation patterns within the pancreas also remain unclear. A previous study revealed that few sympathetic fibres contact the endocrine cells of islets in humans [18]. However, more recent 3D imaging studies suggested significant sympathetic innervation in islets, particularly in contact with α- and δ-cells [ 19– 21]. In the mouse pancreas, a rich supply of sympathetic nerves was found to contact α-cells but appeared not to branch in space to establish contacts with β-cells [ 21, 22]. Impressively, Giannulis *et al*. [23] reported that the islets of genetically obese mice presented increased sympathetic innervation of the pancreatic islets, with increased numbers of TH-positive fibres contacting β-cells. Nevertheless, multiple studies have suggested that a loss of sympathetic nerves occurs in individuals with diabetes [ 24– 27]. In light of these discordant findings, there is a pressing need to comprehensively map pancreatic sympathetic innervation and elucidate its functional significance in glucose homeostasis.

In the present study, we systematically investigated sympathetic innervation in the pancreas of both wild-type (WT) and diabetic mice. We observed that 45.9% of α-cells and 31.8% of β-cells were innervated by sympathetic nerves in 10-week-old WT mice. Moreover, the number of sympathetic innervated α/β-cells was reduced in diet-induced obese (DIO) mice, whereas the number of sympathetic innervated β-cells was elevated in *db*/ *db* mice. Interestingly, unlike the core-mantle structure observed in WT and DIO mice, α-cells and β-cells are intermixed in *db*/ *db* mice. In addition, *in situ* cPSD led to an improvement in glucose tolerance in WT and *db*/ *db* mice but a decrease in DIO mice. Importantly, sympathetic denervation enhanced insulin sensitivity specifically in diabetic mice, with no observable effect on WT mice. These findings underscore the pivotal role of pancreatic sympathetic innervation in glucose homeostasis, providing new insights for a deeper understanding of diabetes.

## Materials and Methods

### Animals

Eight-week-old C57BL/6J mice and leptin-deficient *db*/ *db* male mice were purchased from GemPharmatech Co., Ltd. (Nanjing, China). The DIO mice were established by feeding with a high-fat diet (a rodent diet with 60 kcal% fat, D12492; Shanghai Jinpan Biotech, Shanghai, China) to 10-week-old WT mice over a 16-week period. The mice were acclimated to standard temperature, humidity, and light conditions for 7 days before the experiments. The mice were randomly assigned to 2 groups: the vehicle group and the 6-OHDA group. The animal experiments were approved by the Laboratory Animal Management Committee of ShanghaiTech University (approval number: 20230809001).

### Sympathetic nerve ablation

Mice (WT and *db*/ *db* mice at 10 weeks of age and DIO mice at 26 weeks of age) were anesthetized with isoflurane. A feedback heater was used to keep the mice warm during surgery. The hair in the abdomen close to the pancreas was shaved, and the area was sterilized with 95% ethanol-soaked sterile gauze. A midline incision was made in the targeted area of the abdomen skin (as viewed by the operator) to expose the pancreas, and styptic powder was applied to the area to prevent bleeding. The pancreas was pulled out gently and fully exposed by sterile tweezers. 6-OHDA (H4381; Sigma, St Louis, USA) was dissolved in saline solution containing 0.2% L-ascorbic acid (A92902; Sigma) to achieve a final concentration of 10 mg/mL. To pharmacologically ablate sympathetic nerves in the pancreas, a 20-μL dose of 6-OHDA was evenly administered from the pancreatic head to the tail in the 6-OHDA group, utilizing a LEGATO ^®^ 130 SYRINGE PUMP (#788130; RWD; Kakogawa, Japan). An equivalent volume of 0.2% L-ascorbic acid solution was administered to the vehicle group. The pancreas was then gently placed back into its original position. The mice were allowed to recover in a warm blanket before they were transferred to housing cages. One to two weeks after pharmacological ablation, the mice were subjected to subsequent experimental procedures.

### Intraperitoneal glucose tolerance tests (GTTs) and intraperitoneal insulin tolerance tests (ITTs)

GTTs were conducted in mice after 6 h of fasting following an intraperitoneal (ip) injection of glucose (20% dextrose) at a dose of 2.0 g/kg body weight. Blood samples were collected from the mouse orbital vein at the indicated time points for subsequent analysis via an enzyme-linked immunosorbent assay (ELISA). ITTs were performed in 6-h fasted mice by injecting 0.75 units/kg body weight (for WT mice), 1.0 units/kg (for DIO mice) or 1.5 units/kg (for *db*/ *db* mice) recombinant human insulin (100 U/mL; Novo Nordisk, Shanghai, China).

### Physiological measurements

Glucose was measured via a hand-held glucometer (Accu-Chek Performa Connect; Roche, Basel, Switzerland). Blood samples were collected from the tail vein of ad libitum-fed mice. The concentrations of serum insulin and glucagon were measured via insulin and glucagon ELISA kits (#JL1145 & #JL20654; J&L Biology, Shanghai, China) according to the manufacturer’s instructions.

### Immunohistochemistry

The mice were anesthetized and perfused with phosphate-buffered saline (PBS) followed by 4% paraformaldehyde (PFA). The pancreas was excised and postfixed overnight. The pancreas was dehydrated sequentially in 15% sucrose solution and 30% sucrose solution for 2 days at 4°C and then sectioned at 40-μm thicknesses on a cryostat microtome (CM3050s; Leica, Wetzlar, Germany). The slices were washed three times in PBST (PBS with 0.3% Triton X-100, v/v) and blocked with QuickBlock™ blocking buffer for 2 h at room temperature. Then, the slices were incubated for 36 h with primary antibodies (1:500) diluted in QuickBlock™ primary antibody dilution buffer. After that, the slices were washed 3 times in PBST before being incubated with secondary antibodies (1:1000) for 12 h at room temperature. Pancreatic sections were washed 3 times in PBST, stained with DAPI and cover-slipped for subsequent image processing. The immunohistochemical reagents utilized in this study are detailed in Supplementary Table S1.

### Imaging processing and analysis

High-resolution imaging was performed via a Leica SP8 STED 3X confocal microscope (operated with Leica Application Suite X, version 3.5.2). Excitation was delivered via 405 nm, 488 nm, 568 nm and 633 nm laser lines. The signals were detected at 410–481 nm (DAPI), 498–560 nm (Alexa 488), 578–630 nm (Alexa 568) and 641–739 nm (Alexa 633) via HyD spectral detectors. Confocal images (pinhole = airy 1, step size = 0.5 μm) of randomly selected islets (9–15 islets per section) were acquired on a Leica SP8 confocal microscope. Quantitative analysis was performed via Imaris 9.7.2 (Bitplane AG, Zürich, Switzerland) and Fiji open-source software. A subset of experiments was performed via a Nikon CSU-W1 Sora 2 camera confocal microscope (Nikon, Tokyo, Japan) equipped with a 20× 0.8/air objective.

To calculate the numbers of β-cells and α-cells per slice, each islet was evaluated to obtain the number of cells within this islet that were positive for insulin and glucagon using Imaris version 9.7.2. Imaris software was used to create digital surfaces covering the islets and innervation to automatically determine the volume and intensity data. Volume reconstructions were performed via the surface function with local contrast background subtraction. For the detection of islets, the threshold factor was set to “20”, corresponding to the largest α- and β-cell diameter in each sample, and the automated surface algorithm with a 5-μm “smooth texture” was used to exclude hypointense (non-tissue-filled) regions. The numbers of islets with core α-cells were determined according to a rule reported previously [7]: the edge of the islet was used as the boundary, 2 layers of cells were indented toward the interior of the islet, and the number of α-cells within the circle was quantified.

The nerve fibre plexus was reconstructed in 3D stacks of images. For the detection of nerves, the threshold factor was set to 1.5 μm. In confocal images, digital surfaces were created to cover nerve fibres and individual β-cells and α-cells. The Imaris distance transform MATLAB XTension function was used to calculate the distance of each α- and β-cell surface from the innervation surface. This measurement was subsequently employed to determine the distance between sympathetic nerves and individual α- or β-cells, with a distance of 0 indicating nerve contact [ 20, 28].

### Morphometric measurements

For quantification, slices were digitized with OlyVIA Vs120 software on a fluorescence scan at 20× magnification. Quantitative analysis of the islet area was performed with ImageJ software (NIH, Bethesda, USA) via the positive-pixel count algorithm, expressed in μm ^2^. For the analysis of islet size, we referred to various classification methods [29]. The classification criteria for islets are as follows: Islets with an area exceeding 25,000 μm² are categorized as ‘Large Islets’ (LI), those with an area less than 10,000 μm² are categorized as ‘Small Islets’ (SI), and those in between are categorized as ‘Medium Islets’ (MI). To determine the sizes of islets and counts of α/β-cells, immunostaining of insulin and glucagon was performed in pancreatic sections. This analysis involved manually capturing images and counting the islets. Image analysis was employed to quantify the entire section, followed by the identification of insulin/glucagon-positive areas using Image J software to calculate the islet area. The ‘closed polygon’ tool in Image J software was used to determine the parameters of the islets.

### Quantification and statistical analysis

All the statistical data from individual segmented islets were exported from Imaris to Excel ^®^ (Microsoft ^®^, version 2010), and each α- or β-cell received an individual ID for sorting purposes. All the results are presented as the mean ± SEM for the indicated number of observations. Student’s *t* test was used for two-group comparisons, one-way ANOVA was used for multi-group comparisons, and two-way ANOVA with multiple comparisons was used for groups mixed by time factorial designs. The data were analyzed using Prism 8.0. A *P* value < 0.05 was considered to indicate statistical significance.

## Results

### Distribution of sympathetic nerves in wild-type pancreatic islets

**Figure 1:**
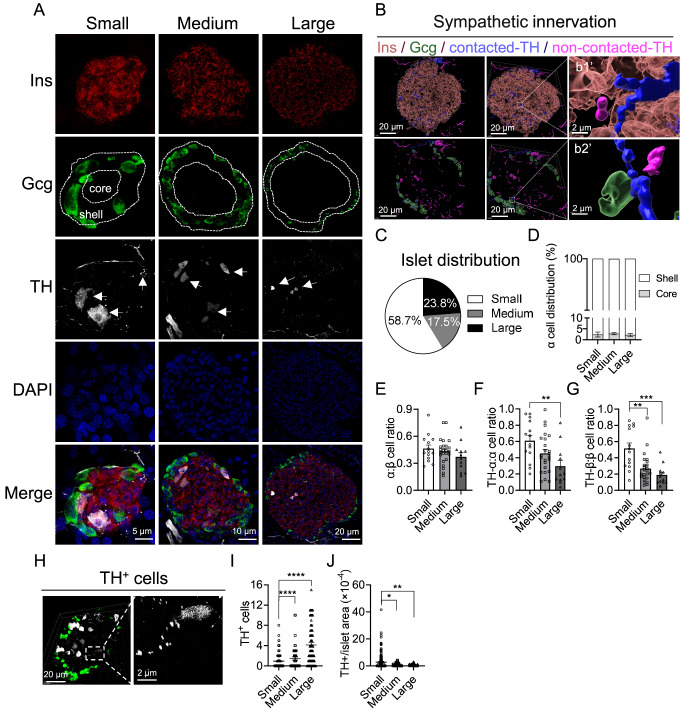
Figure 1 Distribution of sympathetic nerves in wild-type pancreatic islets (A) Immunohistochemical staining for insulin (Ins, red), glucagon (Gcg, green), and tyrosine hydroxylase (TH, gray) in the WT pancreas. The islets exhibit a uniform architecture, with α-cells at the islet periphery surrounding a β-cell core. There are approximately two cell layers between the core and shell dashed lines. The white arrows indicate resident TH+ cells in the islets. (B) Schematic diagram of 3D reconstruction. β-cells (pink, transparent), α-cells (green, transparent), α/β-cells in contact with THs (blue, opaque), and α/β-cells in contact with THs (purple, opaque). (C) Distribution of islets of different sizes (small islets, n = 168; medium islets, n = 51; large islets, n = 70). (D) Distribution of α-cells located in the core and shell of the islets, as defined in (A). (E) The ratio of α to β cell numbers. (F,G) Percentages of TH-innervated α-cells among total α-cells (F) and TH-innervated β-cells among total β-cells (G). (H) Representative images of resident TH+ cells. (I,J) Distribution of TH+ cells in different islets (I) normalized to the islet area (J). Data are presented as the mean ± SEM and were analyzed by one-way ANOVA. *P < 0.05, **P < 0.01, ***P < 0.001, ****P < 0.0001.

To characterize the distribution of sympathetic nerves in the pancreas, we performed immunohistochemical staining for insulin (Ins), glucagon (Gcg) and tyrosine hydroxylase (TH), which indicate β-cells, α-cells and sympathetic nerves, respectively ( Figure 1A). To further explore the detailed spatial distribution, we reconstructed these confocal images ( Figure 1B). Considering the different sizes, we categorized islets into three groups: small islets (SI, area ≤ 10,000 μm ^2^), medium islets (MI, 10,000–25,000 μm ^2^) and large islets (LI, ≥ 25,000 μm ^2^) ( Figure 1A). We detected 58.7% SI, 17.5% MI and 23.8% LI in adult WT mice (10 weeks old, Figure 1C). To better understand the distribution of α-cells in islets, we used the edge of the islet as the boundary, indented 2 layers of cells toward the interior, and quantified the number of α-cells within the circle. Unlike β-cells, which are ubiquitously expressed throughout all islets, most α-cells are within the shells of the SI, MI, and LI ( Figure 1A,D). We also quantified the numbers of α-/β-cells and found that the ratio of α- to β-cells did not change among the different sizes of islets ( Figure 1E). On the basis of the 3D architecture, we classified α/β-cells into TH-innervated cells and un-innervated cells ( Figure 1B and Supplementary Movies S1–S4). We found that 60.9% of α-cells were innervated by TH in the SI, 45.3% in the MI, and 29.5% in the LI ( Figure 1F). Similarly, 51.6% β-cells were innervated by TH in the SI, 26.7% in the MI, and 18.6% in the LI ( Figure 1G). Interestingly, we detected some TH-positive (TH ^+^) cells within the islets ( Figure 1H). Moreover, the total number of these cells increased from the SI to the LI ( Figure 1I). However, when these values were normalized to islet areas, the relative number of these cells decreased from the SI to the LI ( Figure 1J). Overall, we found that the pancreas of WT mice was predominantly composed of smaller islets and that most α-cells were preferentially distributed within the shell of islets. Notably, we observed an inverse correlation between the size of islets and the proportion of α/β-cells innervated by sympathetic nerves, suggesting that the degree of sympathetic innervation per α/β-cell may be attenuated with islet enlargement, which could have functional implications for islet endocrine activity and glucose regulation.

### Pancreatic sympathetic nerves were reduced in DIO mice

**Figure 2:**
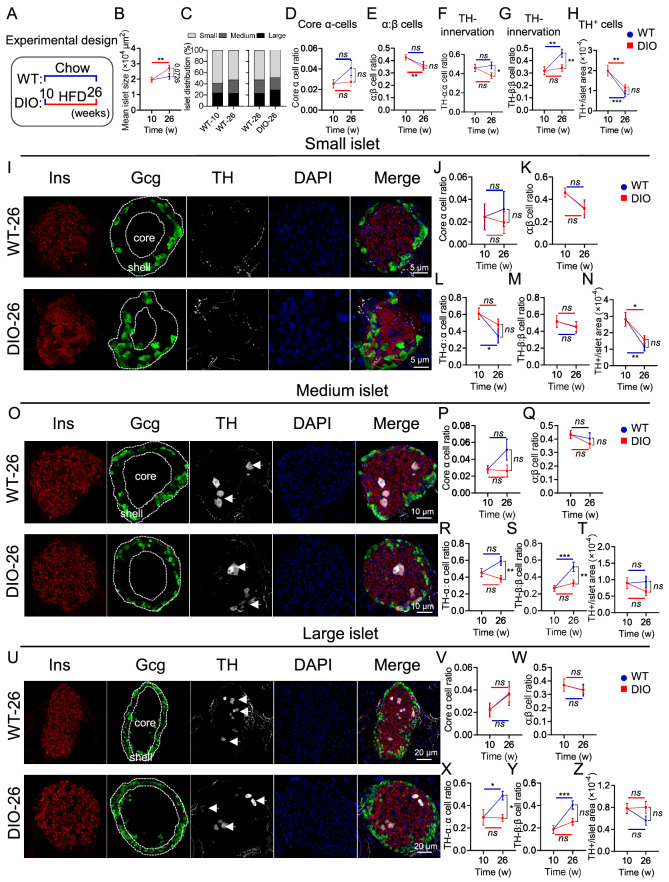
Figure 2 Distribution of sympathetic nerves in the pancreatic islets of DIO mice (A) Experimental design for DIO mice (WT-10/26 refers to WT mice at 10 and 26 weeks of age, respectively; DIO-26 refers to WT-10 mice fed a HFD for 16 weeks). (B,C) Comparison of the mean islet area (B) and islet distribution (C) between WT and DIO mice (WT-10, n = 289 from 4 mice; WT-26, n = 267 from 4 mice; DIO-26, n = 427 from 5 mice). (D) Percentage of α-cells located in the core of the islets. All sizes of islets were quantified. The boundaries are defined in (I), (O) and (U). (E) The ratio of α to β cell numbers for all islets. (F,G) Percentages of TH-innervated α-cells among total α-cells (F) and TH-innervated β-cells among total β-cells (G). (H) Quantification of TH+ cells in all islets. (I) Representative images of small islets in WT-26 mice and DIO-26 mice (WT-10, n = 14; WT-26, n = 13; DIO-26, n = 17). (J) Percentage of α-cells located in the core of small islets. (K) The ratio of α to β cell numbers in small islets. (L,M) Percentages of TH-innervated α-cells among total α-cells (L) and TH-innervated β-cells among total β-cells (M) in small islets. (N) Quantification of TH+ cells in small islets. (O–T) Representative images and quantification of medium islets, with similar quantification as (I–N) (WT-10, n = 24; WT-26, n = 17; DIO-26, n = 24). (U–Z) Representative images and quantification of large islets, with similar quantification as (I–N) (WT-10, n = 12; WT-26, n = 15; DIO-26, n = 19). Data are presented as the mean ± SEM and were analyzed via an unpaired t test. *P < 0.05, **P < 0.01, ***P < 0.001.

To evaluate whether sympathetic innervation is altered in diabetic mice, we induced DIO models by feeding C57 mice with a high-fat diet (HFD) ( Figure 2A). Compared with those of WT-26 mice (WT mice at 26 weeks of age), the islet sizes of DIO-26 mice (DIO mice at 26 weeks of age with a 16-week HFD) were significantly greater ( Figure 2B and Supplementary Figure S1A,B). Compared with WT-10 mice (WT mice at 10 weeks of age), WT-26 mice presented a lower SI percentage (WT-26:WT-10 = 52.9%:58.7%) and an increased MI percentage (WT-26:WT-10 = 24.0%:17.5%) ( Figure 2C). Compared with WT-26 mice, DIO mice presented a decreased SI percentage (DIO:WT-26 = 48.6%:52.9%), MI percentage (DIO:WT-26 = 22.1%:24.0%) and increased LI percentage (DIO:WT-26 = 29.3%:23.1%) ( Figure 2C). Like in WT-26 mice, most α-cells were located within islet shells in DIO mice ( Figure 2D). Moreover, the ratio of α- to β-cells significantly decreased with age in WT mice, but no difference was found between WT-26 and DIO-26 mice ( Figure 2E). We also found no significant difference in TH-innervated α-cells between WT-10 (45.9%) and WT-26 mice (48.5%), whereas the number of TH-innervated α-cells was significantly lower in DIO mice (37.9%) than in WT-26 mice (48.5%) ( Figure 2F). However, we detected a significant increase in TH-innervated β-cells in WT-26 mice (46.1%) compared with those in WT-10 mice (31.8%) ( Figure 2G). Moreover, it was distinctly lower in DIO mice (34.1%) than in WT-26 mice (46.1%) ( Figure 2G). In addition, the normalized number of TH ^+^ cells was lower in WT-26 or DIO-26 mice than in the corresponding control groups (WT-26 vs WT-10, DIO-26 vs DIO-10), whereas there was no significant difference between DIO mice and WT-26 mice ( Figure 2H).

In the SI, the majority of α-cells were located within islet shells in both WT and DIO mice ( Figure 2I,J). We also found that there was no difference in the ratio of α- to β-cells ( Figure 2K), TH-innervated α-cells ( Figure 2L), TH-innervated β-cells ( Figure 2M) and the normalized number of TH ^+^ cells ( Figure 2N) between DIO and WT mice. In MI, the distribution of α-cells in WT and DIO mice was similar to that in the SI ( Figure 2O,P). Moreover, there was no difference in the ratio of α- to β-cells between DIO and WT mice ( Figure 2Q,R). In addition, the number of TH-innervated α-cells was obviously lower in DIO mice (38.0%) than in WT-26 mice (59.0%) ( Figure 2S). Similar results were obtained in the TH-innervated β-cells (DIO:WT-26 = 32.7%:52.0%) ( Supplementary Figure S2). However, there was no difference in the normalized number of TH ^+^ cells ( Figure 2T). In the LI, most α-cells were also located within islet shells in both WT and DIO mice ( Figure 2U,V). There was no difference in the ratio of α- to β-cells between DIO and WT mice ( Figure 2W). Furthermore, the number of TH-innervated α-cells was lower in DIO mice (28.8%) than in WT-26 mice (48.8%) ( Figure 2X). Similar results were obtained in the TH-innervated β-cells (DIO:WT-26 = 25.6%:40.6%; Figure 2Y). Similar to the findings in the SI and MI groups, there was no difference in the normalized number of TH ^+^ cells between the DIO and WT mice ( Figure 2Z).

Overall, we concluded that islets undergo progressive enlargement with aging and elevated blood glucose level. Moreover, a HFD decreases the sympathetic innervation of both α-cells and β-cells.

### Sympathetic-innervated α/β-cells are altered in *db*/ *db* mice

**Figure 3:**
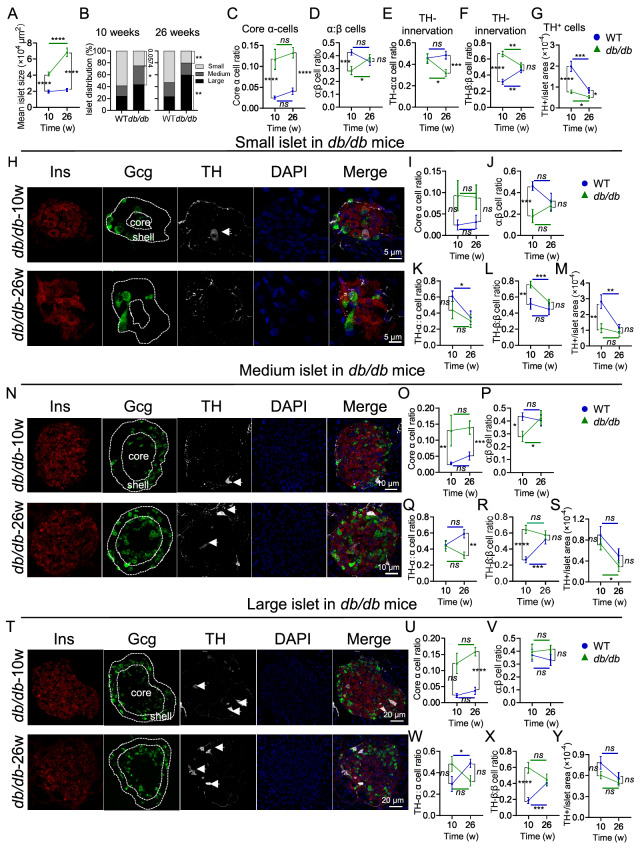
Figure 3 Distribution of sympathetic nerves in the pancreatic islets of *db*/ *db* mice (A,B) Average area and distribution of islets of different sizes in WT mice and db/db mice (WT-10, n = 289 from 4 mice; WT-26, n = 267 from 4 mice; db-10, n = 390 from 3 mice; db-26, n = 256 from 3 mice). (C) Percentage of α-cells located in the core of the islets. (D) The ratio of α to β cell numbers for all islets. (E,F) Percentages of TH-innervated α-cells among total α-cells (E) and TH-innervated β-cells among total β-cells (F) in all islets. (G) Quantification of TH+ cells. (H) Representative images of small islets in db/db mice (WT-10, n = 14; WT-26, n = 13; db-10, n = 10; db-26, n = 10). (I) Percentage of α-cells located in the core of small islets. (J) The ratio of α to β cell numbers for small islets. (K,L) Percentages of TH-innervated α-cells among total α-cells (K) and TH-innervated β-cells among total β-cells (L) in small islets. (M) Quantification of TH+ cells. (N‒S) For medium islets, with similar quantification as in (H–M) (WT-10, n = 24; WT-26, n = 17; db-10, n = 15; db-26, n = 14). (T–Y) For large islets, with similar quantification as in (H–M) (WT-10, n = 12; WT-26, n = 15; db-10, n = 10; db-26, n = 13). Data are presented as the mean ± SEM and were analyzed via an unpaired t test. *P < 0.05, **P < 0.01, ***P < 0.001, ****P < 0.0001.

We further examined diabetic *db*/ *db* mice at 10 weeks of age (db-10) and 26 weeks of age (db-26). The mean islet size of the *db*/ *db* mice was markedly larger than that of the WT mice, especially in the SI and LI ( Figure 3A and Supplementary Figure S2A,B). Compared with those in WT-10 mice (58.7% for SI, 17.5% for MI and 23.8% for LI), the SI percentage (25.0%) in db-10 mice was drastically lower, and the MI percentage (31.6%) and LI percentage (43.4%) were greater ( Figure 3B and Supplementary Figure S2C). Compared with WT-26 mice (52.9% for SI, 24.0% for MI and 23.1% for LI), db-26 mice presented a significantly lower SI percentage (20.1%) and an increased LI percentage (59.9%) but no difference in the MI percentage (20.0%) ( Figure 3B and Supplementary Figure S2C). Unlike islets with a core-mantle structure in WT mice ( Figure 1A), α-cells and β-cells are intermingled in *db*/ *db* mice ( Figure 3C). In contrast to those in WT mice, α-cells in *db*/ *db* mice exhibited a random distribution across the islet area rather than being confined to the shell ( Figure 3C). In addition, the ratio of α:β cells was significantly lower in db-10 mice than in WT-10 mice, but there was no difference between db-26 and WT-26 mice ( Figure 3D). Intriguingly, there was no significant difference in TH-innervated α-cells between WT-10 (45.9%) and db-10 mice (45.3%), whereas it obviously decreased with age in *db*/ *db* mice, and its proportion in db-26 mice (31.9%) was significantly lower than that in WT-26 mice (48.5%) ( Figure 3E). However, we found that the number of TH-innervated β-cells was greater in db-10 mice (66.2%) than in WT-10 mice (31.8%). However, there was no difference between db-26 (52.0%) and WT-26 (46.1%) ( Figure 3F). Moreover, the number of normalized TH ^+^ cells was lower in *db*/ *db* mice than in WT mice regardless of age ( Figure 3G).

In the SI, most α-cells were distributed within the cores of islets in *db*/ *db* mice ( Figure 3H,I). The ratio of α- to β-cells was significantly lower in db-10 mice than in WT-10 mice, and there was no difference between db-26 and WT-26 mice ( Figure 3J). TH-innervated α-cells decreased with age in both WT (WT-26:WT-10 = 34.4%:60.9%) and *db*/ *db* (db-26:db-10 = 30.2%:44.0%) mice. However, there was no difference between WT and *db*/ *db* mice ( Figure 3K). TH-innervated β-cells also decreased with age in both WT mice (WT-26:WT-10 = 44.8%:51.6%) and *db*/ *db* mice (db-26:db-10 = 53.7%:75.5%) ( Figure 3L). Notably, it was significantly greater in *db*/ *db* mice ( Figure 3L). The number of normalized TH ^+^ cells was lower in db-10 mice than in WT-10 mice ( Figure 3M). Compared with that in WT mice, a notable increase in the distribution of α-cells was observed within the core of islets in *db*/ *db* mice ( Figure 3N,O). Moreover, the ratio of α- to β-cells was significantly lower in db-10 mice than in WT-10 mice, and there was no difference between db-26 and WT-26 mice ( Figure 3P). Although there was no difference between db-10 (44.3%) and WT-10 mice (45.3%), the number of TH-innervated α-cells was lower in db-26 mice (32.4%) than in WT-26 mice (59.0%) ( Figure 3Q). TH-innervated β-cells were increased in db-10 mice (64.4%) compared with that in WT-10 mice (26.7%), but there was no difference between db-26 mice (57.5%) and WT-26 mice (52.0%) ( Figure 3R). In addition, there was no difference in the number of normalized TH ^+^ cells between *db*/ *db* mice and WT mice ( Figure 3S). In the LI ( Figure 3T), more α-cells were distributed in the cores of islets in db-26 mice than in the cores of WT-26 mice ( Figure 3U). However, there was no difference in the ratio of α- to β-cells ( Figure 3V), TH-innervated α-cells ( Figure 3W) or normalized TH ^+^ cells ( Figure 3Y) between *db*/ *db* and WT mice. Interestingly, TH-innervated β-cells were increased in db-10 mice (59.7%) compared with that in WT-10 mice (18.75%), but no difference was detected between db-26 mice (44.8%) and WT-26 mice (40.6%) ( Figure 3X).

In summary, we concluded that islets in *db*/ *db* mice exhibit a random distribution of α-cells and β-cells compared with those in WT mice, which have a uniform structure. In addition, there are more sympathetic innervated β-cells in *db*/ *db* mice than in WT mice. These data suggest that the distribution of α-/β-cells and their sympathetic innervation may be involved in the development of diabetes.

### Effect of cPSD on glucose metabolism

**Figure 4:**
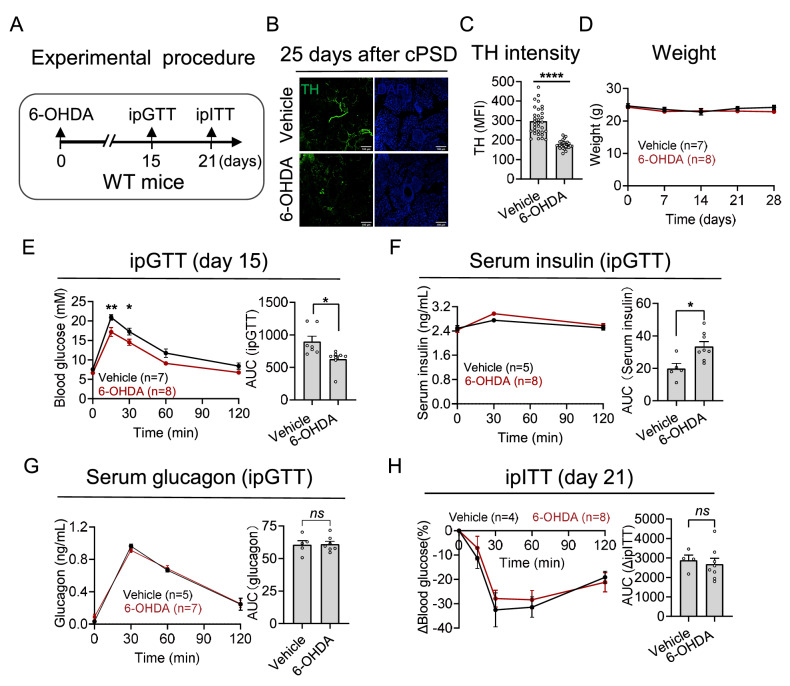
Figure 4 Effects of pancreatic sympathetic denervation in WT mice (A) An experimental design involving multiple injections of 6-OHDA administered at various sites within the pancreas was used to examine the impact of sympathetic denervation on WT mice (10 weeks old). (B) Immunofluorescence staining of TH in the pancreas 25 days after 6-OHDA injection. (C) Quantification of the mean fluorescence intensity (MFI) (normalized to that of the vehicle group) of TH. Scale bar: 100 μm. (D) Changes in body weight after sympathetic denervation. (E) Changes in blood glucose (left) and area under the curve (AUC, right) in WT mice subjected to the intraperitoneal glucose tolerance test (ipGTT). (F) Changes in the serum insulin concentration (left) and AUC (right) during the ipGTT. (G) Changes in the serum glucagon concentration (left) and AUC (right) during the ipGTT. (H) Changes in blood glucose (left) and the AUC (right) of WT mice subjected to the intraperitoneal insulin tolerance test (ipITT). Data are presented as the mean ± SEM and were analyzed by two-way ANOVA with multiple comparisons and unpaired t tests. *P < 0.05, **P < 0.01, ****P < 0.0001.

To examine the role of sympathetic innervation in glucose metabolism, we first executed chemical denervation in the pancreas of WT mice *in situ*. To achieve this goal, we administered 6-hydroxydopamine (6-OHDA) into the pancreas to ablate the sympathetic nerves ( Figure 4A). TH expression was significantly reduced at 25 days after 6-OHDA administration ( Figure 4B,C). Although there was no change in body weight, glucose tolerance improved 15 days after 6-OHDA injection, as determined by the intraperitoneal glucose tolerance test (GTT) ( Figure 4D,E). We also measured insulin ( Figure 4F) and glucagon ( Figure 4G) concentration during the GTT, and a significant increase in the accumulation of insulin was observed ( Figure 4F), implying that sympathetic denervation increases insulin secretion. However, sympathetic denervation had no effect on insulin sensitivity in WT mice, as measured by the intraperitoneal insulin tolerance test (ITT) ( Figure 4H).

**Figure 5:**
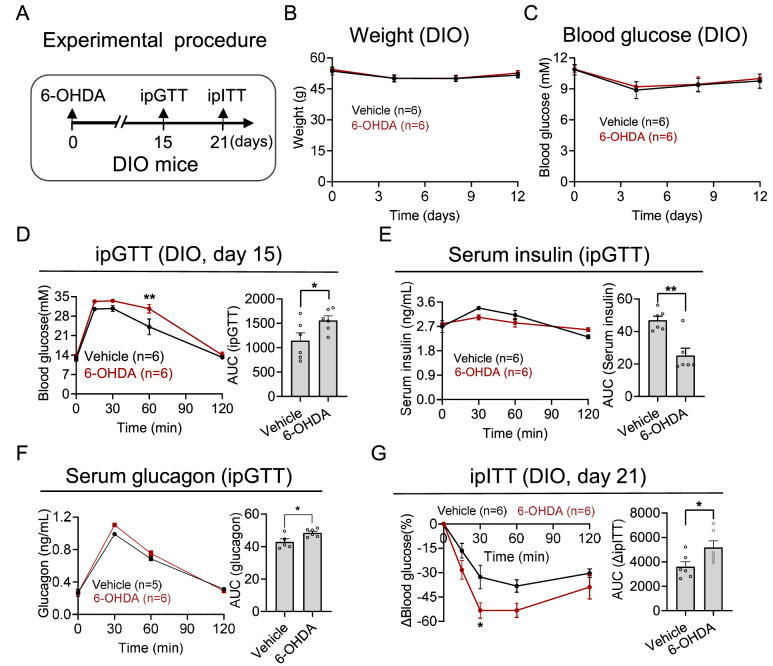
Figure 5 Effects of pancreatic sympathetic denervation in DIO mice (A) An experimental design involving multiple injections of 6-OHDA administered at various sites within the pancreas was implemented to examine the impact of sympathetic denervation on DIO mice. (B,C) Changes in body weight (B) and blood glucose (C) after sympathetic denervation. (D) Changes in blood glucose (left) and the AUC (right) of DIO mice subjected to ipGTTs. (E) Changes in the serum insulin concentration (left) and AUC (right) during the ipGTT. (F) Changes in the serum glucagon concentration (left) and AUC (right) during the ipGTT. (G) Changes in blood glucose (left) and the AUC (right) of DIO mice subjected to ipITT. Data are presented as the mean ± SEM and were analyzed via two-way ANOVA with multiple comparisons and unpaired t tests. *P < 0.05, **P < 0.01.

Next, we injected 6-OHDA into the pancreas of DIO mice ( Figure 5A). We detected no difference in body weight or blood glucose between the 6-OHDA and vehicle groups ( Figure 5B,C). However, glucose tolerance deteriorated after 6-OHDA injection ( Figure 5D). Moreover, the accumulation of insulin was reduced, and glucagon was increased in the 6-OHDA group compared with those in the vehicle group ( Figure 5E,F). Furthermore, the DIO mice exhibited increased insulin sensitivity 21 days after the 6-OHDA injection, as determined by the ITT ( Figure 5G).

**Figure 6:**
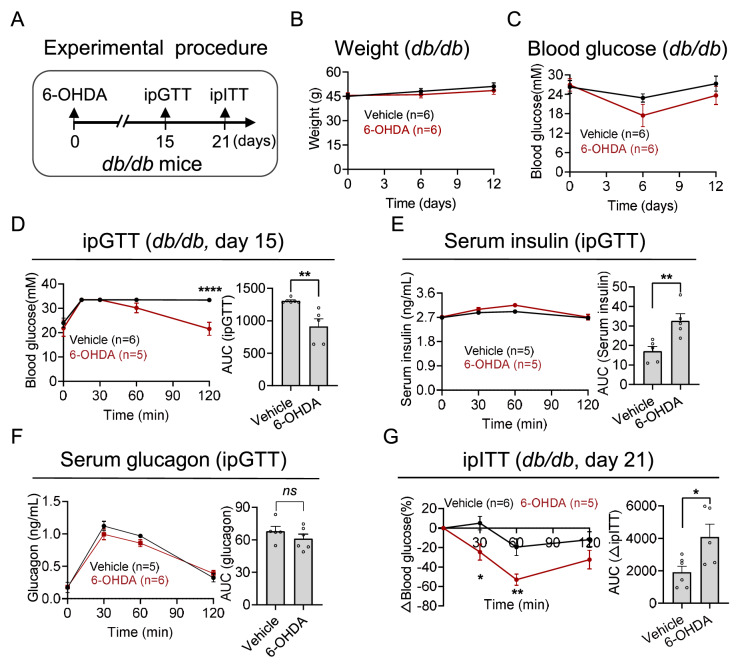
Figure 6 Effects of pancreatic sympathetic denervation in *db*/ *db* mice (A) An experimental design involving multiple injections of 6-OHDA administered at various sites within the pancreas was used to examine the impact of sympathetic denervation on db/db mice (10 weeks old). (B,C) Changes in body weight (B) and blood glucose (C) after sympathetic denervation in db/db mice. (D) Changes in blood glucose (left) and the AUC (right) of db/db mice subjected to ipGTTs. (E) Changes in the serum insulin concentration (left) and AUC (right) during the ipGTT. (F) Changes in the serum glucagon concentration (left) and AUC (right) during the ipGTT. (G) Changes in blood glucose (left) and the AUC (right) of db/db mice subjected to ipITT. Data are presented as the mean ± SEM and were analyzed via two-way ANOVA with multiple comparisons and unpaired t tests. *P < 0.05, **P < 0.01, ****P < 0.0001.

We also measured cPSD in *db*/ *db* mice ( Figure 6A). Compared with those in the vehicle group, neither body weight nor blood glucose was altered after 6-OHDA injection ( Figure 6B,C). Notably, *db*/ *db* mice injected with 6-OHDA exhibited increased glucose tolerance during the GTT ( Figure 6D). Consistently, there was an increase in the accumulation of insulin secretion in response to glucose challenge ( Figure 6E). However, there was a discernible downwards trend in the accumulation of glucagon ( Figure 6F). Moreover, insulin sensitivity significantly increased 21 days after 6-OHDA injection ( Figure 6G).

Taken together, cPSD improves glucose tolerance in WT and *db*/ *db* mice but decreases glucose tolerance in DIO mice. Moreover, cPSD enhances insulin sensitivity in diabetic mice but has no effect on WT mice. These results suggest that pancreatic sympathetic innervation plays an important role in glucose metabolism, which is dependent on physiological or pathological conditions.

## Discussion

Sympathetic innervation and morphological changes in α/β-cells play pivotal roles in islet’s involvement in blood glucose regulation. Nevertheless, the intricate patterns of sympathetic innervation within islets remain poorly understood. To address this gap in knowledge, we meticulously analyzed the sympathetic innervation patterns in islets and their temporal dynamics via high-resolution imaging and advanced 3D reconstruction techniques in adult WT, DIO, and *db*/ *db* mice. Here, we offer a unique opportunity to identify the anatomical and functional features of pancreatic sympathetic innervation in mice under different physio-pathological conditions, providing a new strategy to improve diabetes. Accumulating evidence from laboratory and human studies suggests that therapeutically targeting sympathetic overactivity could help to prevent metabolic diseases [30]. Like the established use of renal sympathetic denervation in clinical practice for humans [31], we look forward to implementing sympathetic intervention techniques for the management of diabetes and other pancreatic-related diseases.

Employing advanced imaging methodologies, we successfully delineated distinctive alterations in the distribution and composition of α/β-cells and sympathetic innervation within the pancreatic islets of WT and diabetic mice (Figures 1– 3). In our study, we categorized islet size via systematic classification methods [ 29, 32]. By integrating data pertaining to islet size alongside its cellular constituents, we were able to provide a more nuanced depiction of the intrinsic microcellular milieu of the islet. We found that small islets constituted the majority of the pancreas of WT mice, and the percentage of small islets decreased significantly with aging ( Figure 2C). In addition, a tendency towards a decrease in islet size was noted in DIO mice ( Figure 2C), and a significant reduction was observed in *db*/ *db* mice ( Figure 3B). Taken together, our findings suggest a propensity for pancreatic islets to undergo gradual enlargement with aging and elevated blood glucose level, corroborating a previous report [33] **.** This increase may originate from the expansion of the β-cell mass, potentially initiated by downstream signaling of the insulin receptor or indirectly modulated through the regulation of peroxisome proliferator-activated receptor-gamma (PPAR-γ) [ 34, 35]. Meanwhile, our findings revealed a positive correlation between aging or HFD consumption and the enlargement of pancreatic islets. These findings suggest that an increase in the number of larger islets may be associated with a decline in cellular function, which is consistent with previous reports [ 36, 37]. Moreover, we noted that the ratio of α- to β-cells decreased with aging in both WT and DIO mice ( Figure 2E) but increased in *db*/ *db* mice ( Figure 3D), indicating desynchronization in the alterations of α-cells and β-cells as the islets enlarge.

In WT mice, pancreatic islets exhibit a distinctive arrangement in which β-cells are predominantly located in the core of islets and are surrounded by α-cells in the shell ( Figure 1A), which is consistent with prior reports [ 7, 38, 39]. With increasing age in mice, the number of α-cells within the core region increases ( Figure 2D). Interestingly, a distinct distribution pattern of α-cells and β-cells was evident in the islets of *db*/ *db* mice ( Figure 3C). Unlike the core-mantle structure observed in WT and DIO mice ( Figures 1A and 2D), α-cells and β-cells are intermixed in *db*/ *db* mice ( Figure 3C). This unique distribution, similar to that in humans [ 11, 39, 40], has implications for the distinct functional role of α/β-cells under diabetic conditions. Notably, there was an increase in the distribution of α-cells within the core of islets in *db*/ *db* mice, suggesting a potential compensatory mechanism in response to the diabetic milieu. Our findings enhance the understanding of the intricate organization and functional implications of pancreatic islets in different genetic backgrounds and under different dietary conditions. This comprehensive analysis contributes to the improvement of clinical protocols for islet transplantation, thereby propelling progress across a spectrum of endeavors in the fields of islet and pancreatic research and therapy.

Compared with that in WT-10 mice, the proportion of TH-innervated β-cells to total β-cells was greater in WT-26 mice ( Figure 2G), indicating that aging is a factor influencing sympathetic innervation in islets. In DIO mice, both TH-innervated α-cells and β-cells were reduced ( Figure 2F,G), suggesting that a HFD affects pancreatic sympathetic nerves. However, although the number of TH-innervated α-cells decreased ( Figure 3E), the number of TH-innervated β-cells increased in *db*/ *db* mice ( Figure 3F). A previous study reported an inclination towards increased noradrenergic innervation of the endocrine area in obese *db*/ *db* mice that aged as diabetes progressed [23]. This discrepancy could be attributed to factors such as excessively thin section thickness, outdated imaging modalities, and suboptimal software quantification. Our data revealed that islet α/β-cells presented relatively abundant sympathetic innervation in both WT and diabetic mice. Furthermore, our study profoundly reinforces the notion that the sympathetic innervation of mouse islet β-cells is conspicuously elevated in comparison with prior reports [ 21, 22], thereby carrying momentous implications for sampling accuracy and cellular functionality.

In our histomorphological study, we observed TH-expressing cells within islets, which were reduced in diabetic mice. Notably, some of the TH ^+^ cells presented distinctive dendritic structures ( Figure 1H), establishing contacts with endocrine cells. As reported previously [ 41– 44], some β-cells express TH, which is crucial for insulin secretion in mice. Hence, these factors should be taken into consideration in future studies exploring these innervations and their functions.

Borden *et al*. [7] reported that no defects in glucose tolerance were detected in sympathectomized mice, but Jimenez-Gonzalez *et al*. [45] reported that the activation of sympathetic neurons impaired glucose tolerance. We found that glucose tolerance was greatly improved in WT mice with pancreatic sympathetic denervation via *in situ* injection of 6-OHDA ( Figure 4E). Notably, both glucose tolerance and insulin sensitivity were significantly improved in *db*/ *db* mice ( Figure 6D,G). Additionally, there was a marked increase in the insulin sensitivity of DIO mice ( Figure 5G). The only discrepancy is that sympathetic denervation decreases glucose tolerance in DIO mice ( Figure 5D). The opposite effect on the ipGTTs in the DIO and *db*/ *db* mice might be attributed to genetic background, as the *db*/ *db* mice are leptin receptor deficient.

In this study, we explored the roles of pancreatic sympathetic nerves under physiological and diabetic conditions, suggesting the potential of manipulating pancreatic sympathetic nerves as a novel therapeutic approach for T2D. However, given that both α-cells and β-cells in the pancreas are innervated by sympathetic nerves, selectively manipulating nerves that target only α-cells or β-cells is challenging. Further studies should focus on in-depth analyses of the molecular profiles, cellular properties, and dynamic roles of sympathetic nerves.

In summary, our findings provide insights into the impact of sympathetic innervation on blood glucose regulation under different physio-pathological conditions. At present, most anti-diabetic drugs regulate blood sugar by enhancing the secretion of insulin or improving insulin sensitivity. Our results suggest that pancreatic sympathetic denervation can improve insulin sensitivity in both DIO and *db*/ *db* mice. We believe that pancreatic sympathetic modulation might become a novel therapeutic strategy for T2D, resembling renal denervation for patients with hypertension [31].

## Supplementary Data

Supplementary data is available at *Acta Biochimica et Biophysica Sinica* online.
